# Influence of adiponectin and inflammatory cytokines in fatty degenerative atrophic muscle

**DOI:** 10.1038/s41598-022-05608-x

**Published:** 2022-01-28

**Authors:** Issei Shinohara, Takeshi Kataoka, Yutaka Mifune, Atsuyuki Inui, Ryosuke Sakata, Hanako Nishimoto, Kohei Yamaura, Shintaro Mukohara, Tomoya Yoshikawa, Tatsuo Kato, Takahiro Furukawa, Takehiko Matsushita, Ryosuke Kuroda

**Affiliations:** Department of Orthopedic Surgery, Graduate School of Medicine, Kobe University, 5-2, Kusunoki-cho7, Chuo-ku, Kobe-shi, Hyogo, 650-0017 Japan

**Keywords:** Cell biology, Pathogenesis

## Abstract

Tendon rupture and nerve injury cause fatty infiltration of the skeletal muscle, and the adipokines secreted from the infiltrated adipocytes are known to contribute to chronic inflammation. Therefore, in this study, we evaluated the effects of the adipokines on chronic inflammation using a rat sciatic nerve-crushed injury model. In vitro and in vivo experiments showed that the expression of adiponectin was decreased (0.3-fold) and the expression of *Il6* (~ 3.8-fold) and *Tnf* (~ 6.2-fold) was increased in the nerve-crushed group compared to that in the control group. It was also observed that the administration of an adiponectin receptor agonist decreased the levels of *Il6* (0.38-fold) and *Tnf* (0.28-fold) and improved cellular viability (~ 1.9-fold) in vitro. Additionally, in the fatty infiltrated skeletal muscle, low adiponectin levels were found to be associated with chronic inflammation. Therefore, the local administration of adiponectin receptor agonists would prevent chronic inflammation.

## Introduction

Muscle volume is an important factor determining muscle function^1^. Muscle atrophy is characterized by a decrease in the cross-sectional area of muscles, resulting in decreased muscle strength or endurance, or both^2^. Additionally, muscle atrophy that is caused by a wide range of factors, such as tendon rupture or nerve damage-inducing fat infiltration into atrophied muscles, leads to a decrease in mobility, a deterioration of the quality of life, and reduced life expectancy^3,4^. The condition in which fat infiltrates atrophied muscle causing muscle dysfunction is called fatty degeneration^5^. Even though tendon rupture and nerve injuries have been repaired successfully, fatty degeneration is irreversible in some cases^6^.

Adipokine is a collective term of various cytokines secreted from adipose tissues^7^. Among them, inflammatory cytokines, such as interleukin-6 (*IL6*) and tumor necrosis factor (*TNF*), which are secreted by adipocytes infiltrating the muscle, have been identified as factors that enhance inflammation as well as chronic pain^8^. Conversely, adiponectin, which is one of the adipokines, enhances fatty acid oxidation, insulin sensitivity, and glucose uptake, while inhibiting hepatic gluconeogenesis and exerting anti-inflammatory effects^9^. Adiponectin was first identified as a circulating adipokine in plasma; however, it was demonstrated that it originates from skeletal muscles^10,11^. The level of adiponectin in muscle does not seem to be related to circulating levels, highlighting the possibility of an independent process in skeletal muscles^10^. Furthermore, it is also attracting increasing attention owing to its role as a locally expressed paracrine/autocrine factor^11^, and reportedly, its administration can reduce muscle damage^12^. However, the expression of adiponectin and its receptors in muscles after muscle atrophy and fatty infiltration has not been sufficiently studied. Muscle atrophy and fatty infiltration are known to cause chronic inflammation^8^, and we hypothesized that decrease of adiponectin expression is related to expression of inflammatory cytokines after the nerve injury. Therefore, in this study, we evaluated the expressions of adipokines on chronic inflammation using a rat sciatic nerve-crushed injury model^13^. In addition, few reports have shown the effect of adiponectin receptor agonist administration on muscle atrophy and fatty infiltration. We also evaluated the effects of AdipoRon, an adiponectin receptor agonist, on myotubes after the nerve injury in vitro.

## Results

### Gastrocnemius muscle weight

**Figure 1:**
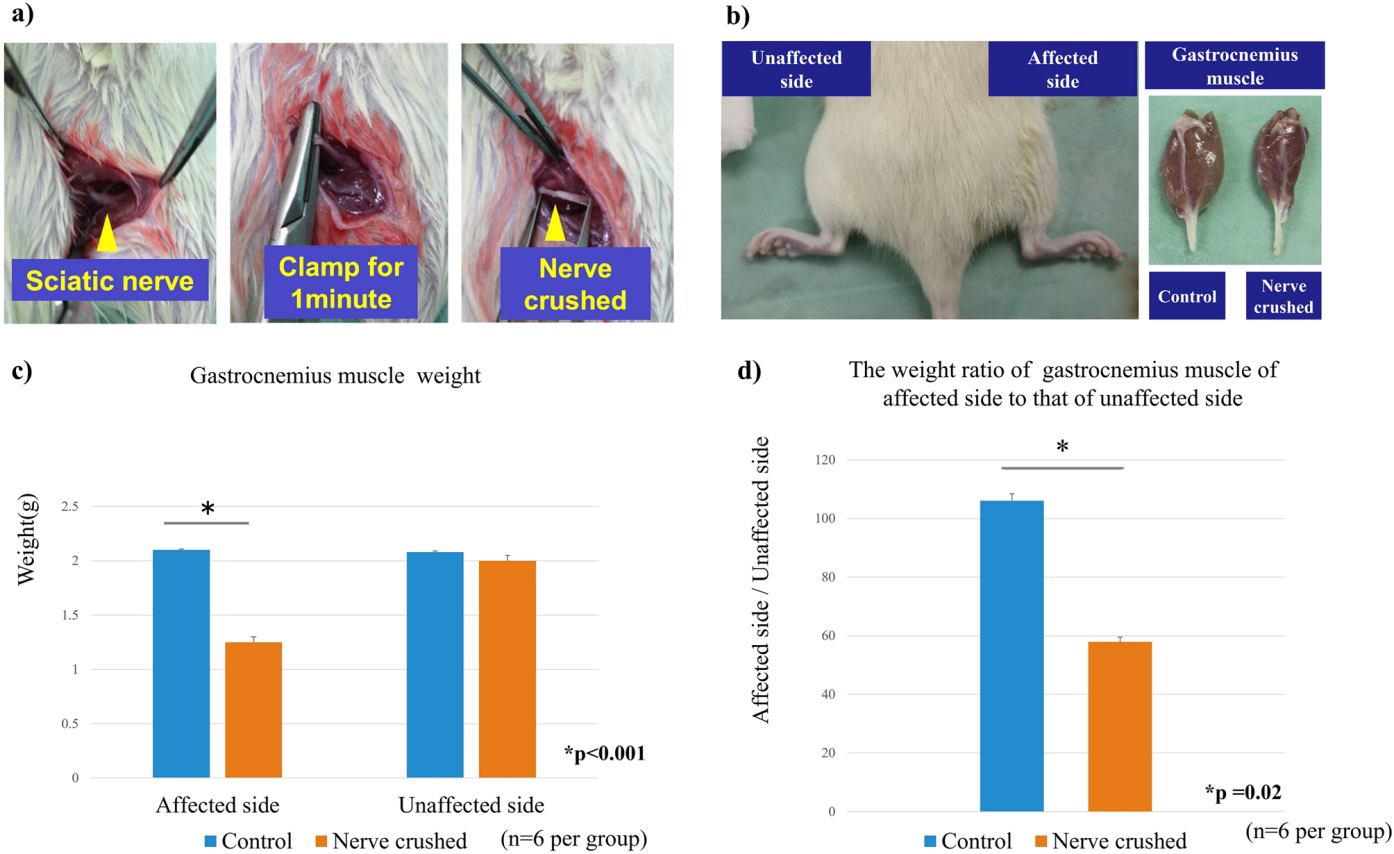
(**a**) Procedure for establishing the nerve-crushed rat model. The sciatic nerve was clamped using hemostatic forceps for one minute at a proximal distance of 5 mm from the bifurcation point of the peroneal and tibial nerves. (**b**) Resected gastrocnemius muscle. (**c**) The weight of the gastrocnemius muscle on the affected side was significantly decreased in the nerve-crushed group compared to the unaffected side (*p* < 0.001). (**d**) The weight ratio of the muscle on the affected side and the healthy side in the nerve-crushed group (57.9 ± 2.3%).

The weight of the gastrocnemius muscle at 4 weeks post-operation was compared between the nerve-crushed model (refer to the Methods section, Fig. 1a, b), in which the sciatic nerve was crushed as described in a previous study^13^, and the control group, which had sham surgery wherein only a skin incision was made. The gastrocnemius muscle weight was significantly decreased on the affected side in the nerve-crushed group than on the unaffected side. The weight of the gastrocnemius muscle in the control group was not significantly different from those of the affected and unaffected sides. There was no significant difference in gastrocnemius muscle weight between the unaffected side of the nerve-crushed group and both sides of the control group (Fig. 1c). The ratio of the muscle weight on the affected side to that on the unaffected side in the nerve-crushed group was significantly lower than that in the control group, as shown in Fig. 1d.

### Cell morphology evaluation

**Figure 2:**
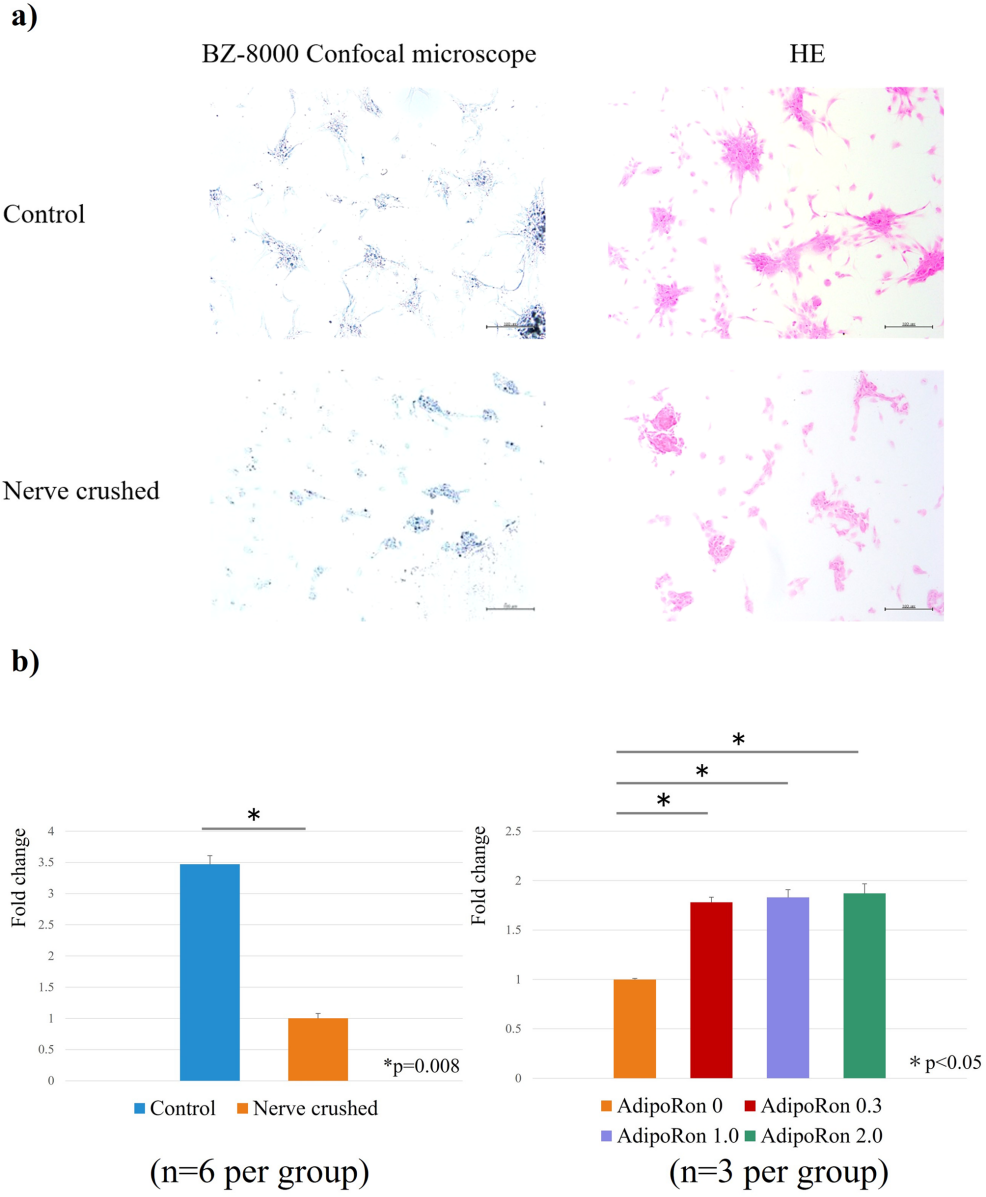
(**a**) Morphological observation based on microscopy and HE staining. The nerve-crushed group showed smaller cells and poorer myotube formation. (**b**) Fold change comparison (baseline: nerve-crushed group). Water-soluble tetrazolium salt (WST) assay showed that myocyte proliferation was significantly lower in the nerve-crushed group than in the control group (*p* = 0.08). AdipoRon treatment significantly improved cell viability in the nerve-crushed group (*p* < 0.05). The different AdipoRon doses showed no significant differences.

Morphological observation via microscopy and Hematoxylin–eosin (HE) staining showed the formation of myotubes in the control group. Conversely, in the nerve-crushed group, the cells appeared small, with poorly formed myotubes (Fig. 2a). The number of myotubes formed per field of view (400 μm × 600 μm) was calculated as the average of four fields of view. The number of myotubes formed per field of view was 24.8 ± 2.3 in the control group and 6.6 ± 1.9 in the nerve-crushed group, and the number of myotubes formed was significantly lower in the nerve-crushed group (*p* = 0.0003).

### Cell viability (cell proliferation assay)

The water-soluble tetrazolium salt (WST) assay was used to measure the absorbance of reduced formazan at 450 nm. Thus, it was observed that the absorbance corresponding to the control group was significantly higher than that corresponding to the nerve-crushed group. Further, in the nerve-crushed group treated with AdipoRon, the absorbance showed a significant increase compared with the nerve-crushed group without AdipoRon treatment. However, the differences among all the AdipoRon doses were not significant (Fig. 2b).

### Fluorescent immunostaining

**Figure 3:**
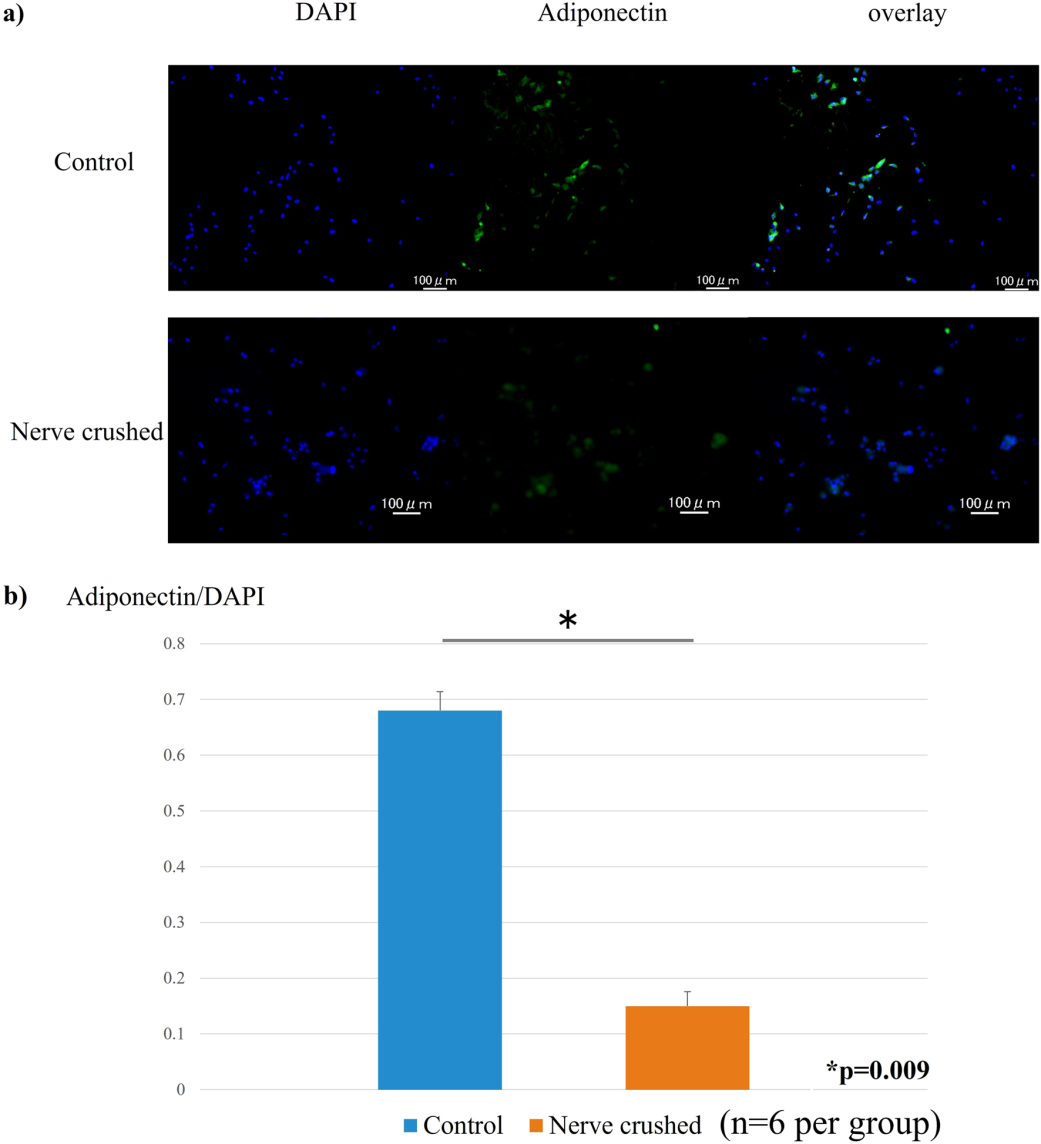
(**a**) Ratio of adiponectin staining evaluated based on the percentage of green adiponectin-stained cells relative to blue-stained DAPI-positive cells. (**b**) The ratio of green-stained adiponectin cells to blue-stained DAPI-positive cells (significantly lower in the nerve-crushed group (0.15) than in the control group (0.68)).

The cytoplasm of the adiponectin-positive cells was stained green, and the quantitative evaluation of the ratio of adiponectin-stained cells to blue-stained 4’,6-diamidino-2-phenylindole (DAPI)-positive cells was determined (Fig. 3a). Thus, we observed that the ratio of green adiponectin-stained cells to blue DAPI-positive cells. Evidently, that corresponding to the nerve-crushed group was significantly lower than that corresponding to the control group (Fig. 3b).

### Histological examination

**Figure 4:**
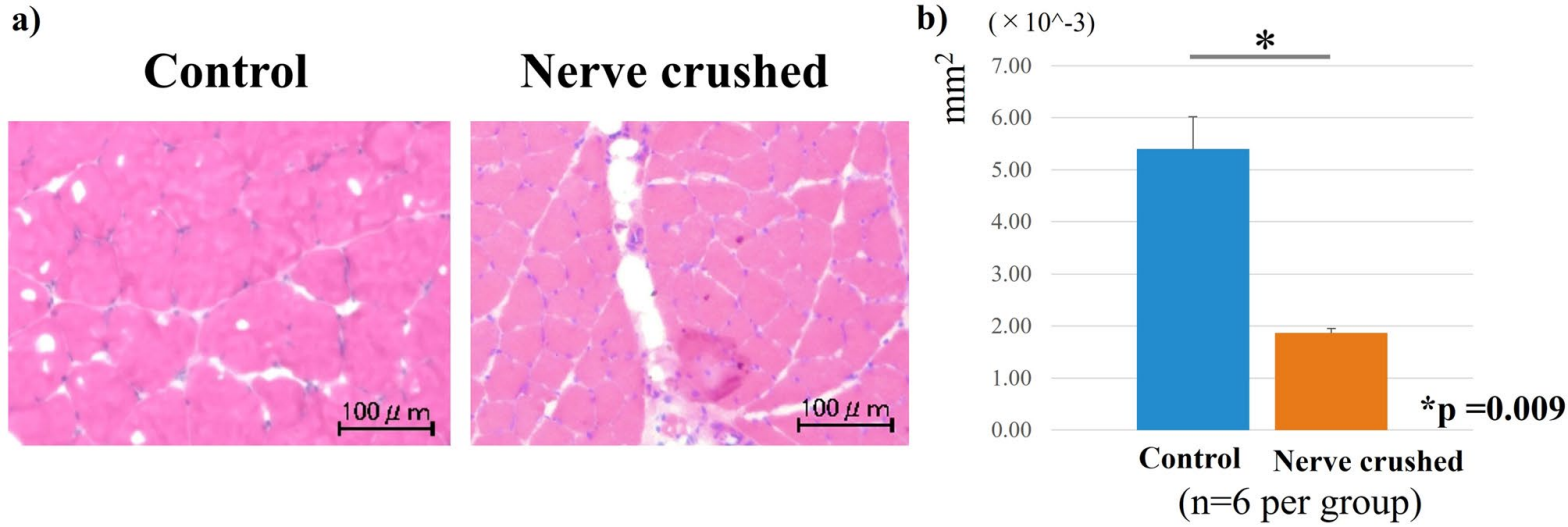
(**a**) Hematoxylin–eosin (HE) staining showing a smaller diameter for the muscle fibers corresponding to the neurolysis group than those corresponding to the control group. (**b**) The mean cross-sectional area of each muscle fiber was significantly reduced in the crushed nerve group compared to the control group. (*p* = 0.009).

**Figure 5:**
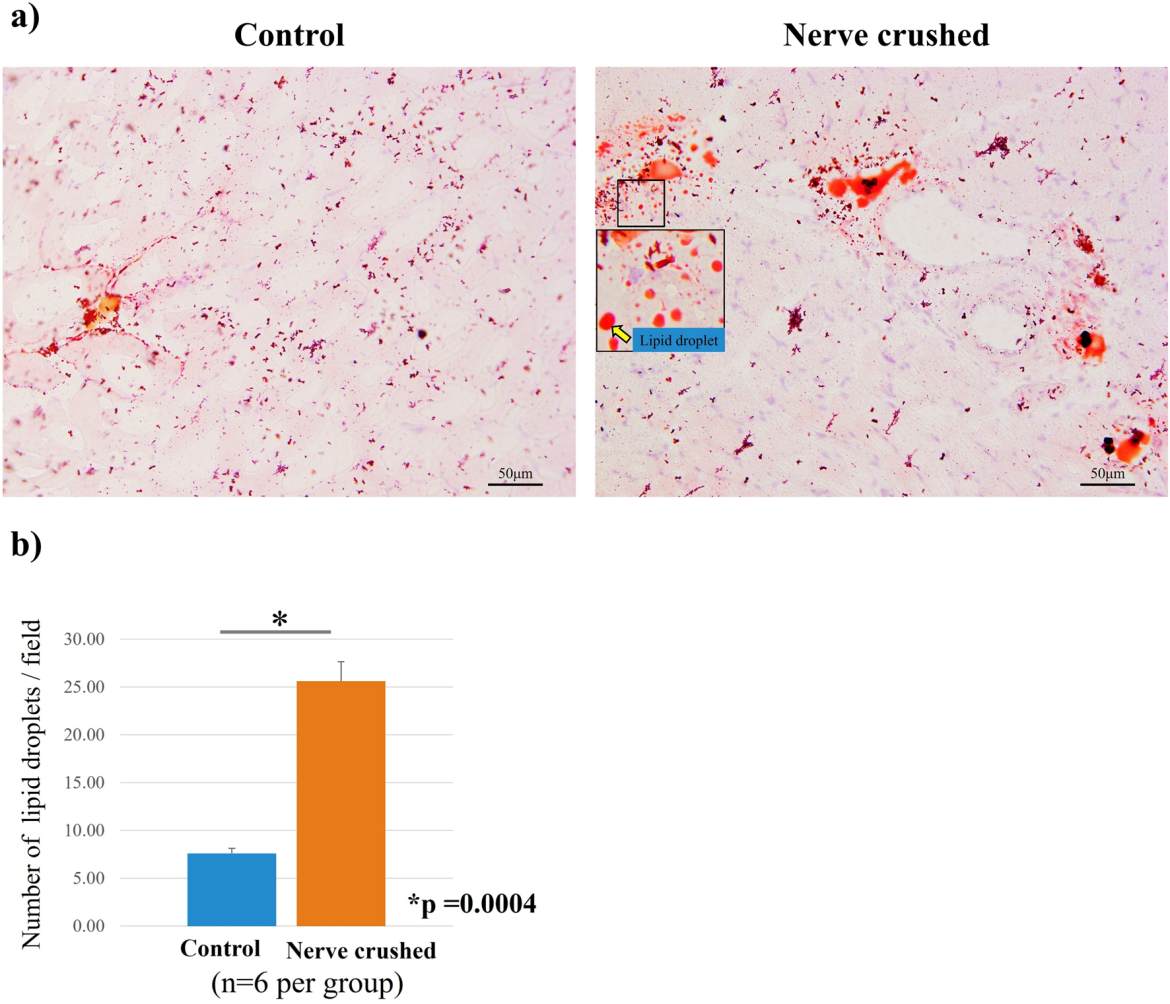
(**a**) Oil red-O staining showed infiltration of fat droplets in the muscle. (**b**) The quantitative assessment of the number of fat droplets showed a significant increase in the nerve-crushed group (*p* = 0.0004).

**Figure 6:**
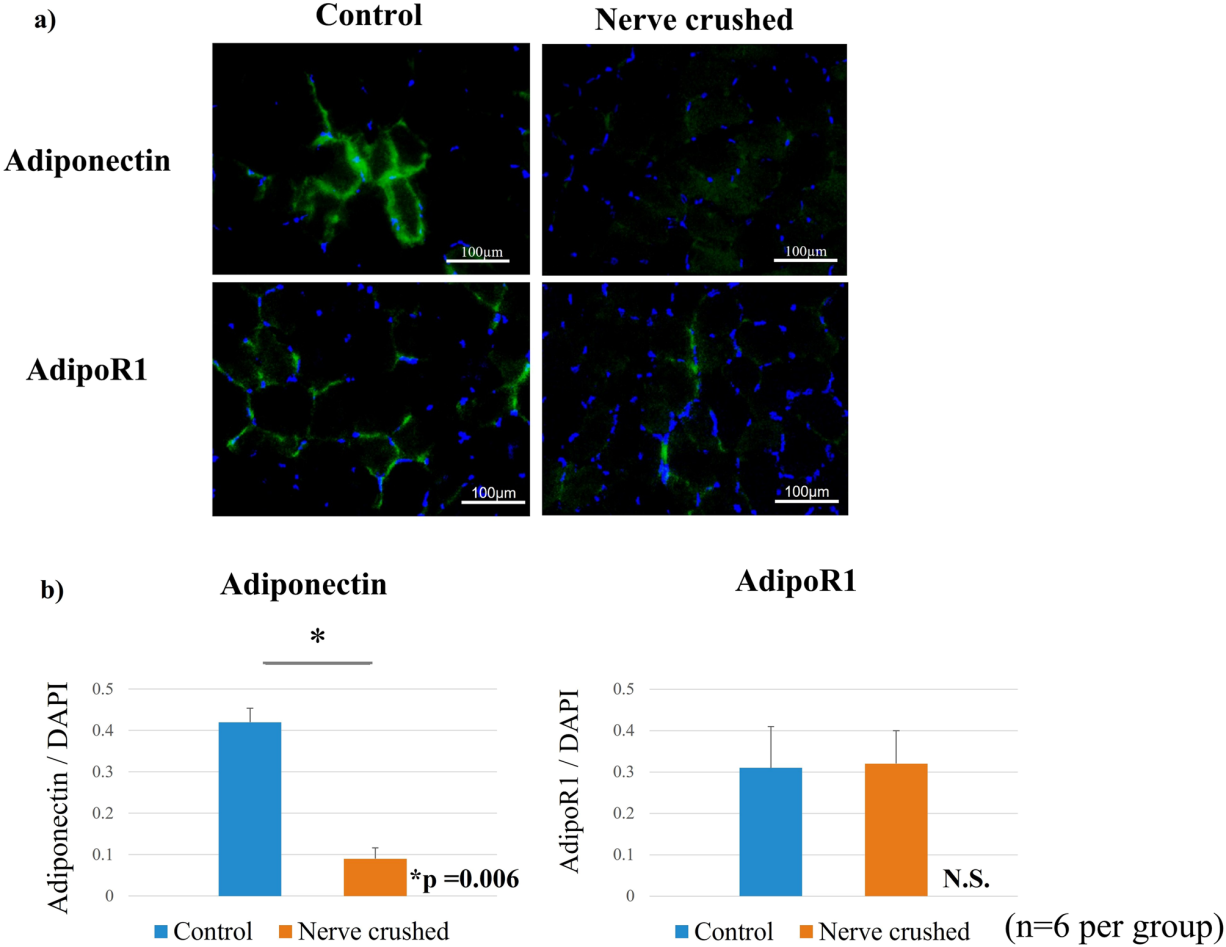
(**a**) Adiponectin and AdipoR1 stained along the fascia. (**b**) Quantitative analysis of adiponectin expression showed that adiponectin expression was significantly lower in the nerve-crushed group (*p* < 0.01) than in the control group. Conversely, AdipoR1 expression showed no significant difference between the two groups.

HE staining showed that the diameter of muscle fibers corresponding to the rats in the nerve-crushed group was smaller than that corresponding to the rats in the control group. Furthermore, the mean cross-sectional area of each muscle fiber was significantly decreased in the nerve-crushed group, indicating that the muscles were atrophied four weeks after the nerve crush injury (Fig. 4a, b). Oil Red-O positive lipid droplets were observed in the muscle in both the control and nerve-crushed groups. The number of fat droplets quantitatively assessed in four non-overlapping areas by microscopy was significantly higher in the nerve-crushed group than in the control group (Fig. 5a, b). Furthermore, immunofluorescence staining showed that adiponectin was stained along the fascia, and the degree of staining corresponding to the nerve-crushed group was lower than that corresponding to the control group (Fig. 6a). Quantitative evaluation performed using the ratios of green adiponectin- and adiponectin receptor (AdipoR)-stained cells to blue DAPI-positive cells showed that the expression of adiponectin was significantly weaker in the nerve-crushed group than in the control group. Conversely, the two groups showed no significant differences with respect to the expression of AdipoR1 (Fig. 6b).

### Real-time PCR

**Figure 7:**
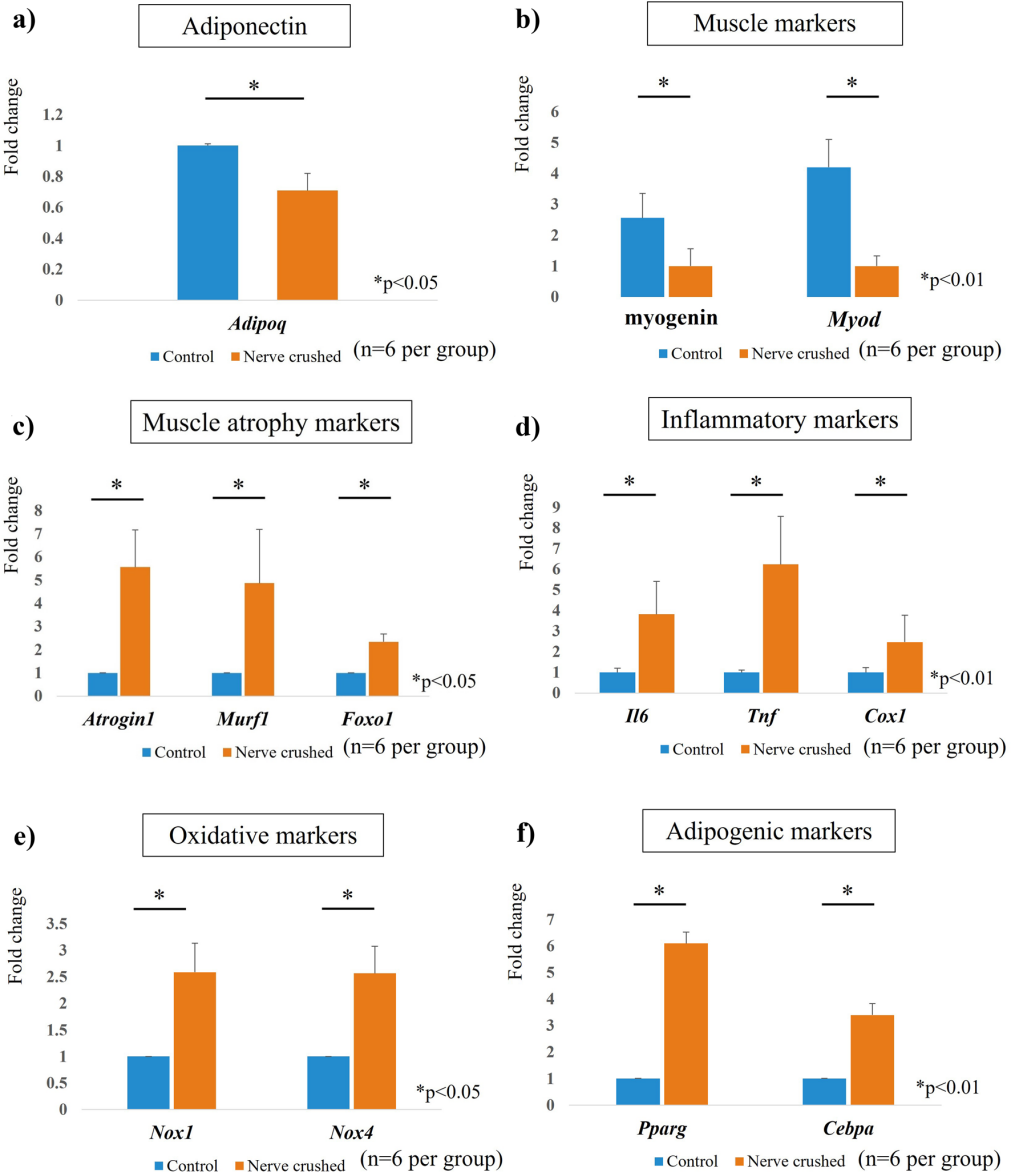
(**a**) *Adipoq* expression was significantly decreased in the nerve-crushed group. (**b**) Gene expression of muscle markers (myogenin and *Myod*; significantly lower in the nerve-crushed group than in the control group) and (**c**) muscle atrophy markers (*Atrogin1*, *Murf1*, *Foxo1*; significantly higher in the nerve-crushed group than in the control group). (**d**) Gene expression of inflammation markers, *Il6*, *Tnf*, and *Cox1*, (**e**) oxidative markers, *Nox1* and *Nox4*, and (**f**) adipogenic markers, *Pparg* and *Cebpa*. (significantly higher in the nerve-crushed group than in the control group).

Expression of the adiponectin (*Adipoq*) gene was significantly decreased in the nerve-crushed group. The gene expression levels of muscle markers, such as myogenin and *Myod*, were significantly lower in the nerve-crushed group, which also showed significantly higher expression levels of muscle atrophy markers (*Atrogin1*, Muscle RING-Finger Protein-1 (*Murf1*), Forkhead box O1 (*Foxo1*)) than the control group. Furthermore, the gene expression levels of *Il6*, *T*nf, and cyclooxygenase1 (*Cox1*) were significantly higher in the nerve-crushed group than in the control group, indicating the occurrence of an inflammatory reaction in the muscles after nerve injury. The expression of oxidative markers, NADPH oxidase1 *(Nox1)* and *Nox4*, was significantly increased in the nerve-crushed group. Furthermore, the expression levels of adipogenic markers, such as *Pparg* and *Cebpa* were significantly higher in the nerve-crushed group than in the control group, as shown in Fig. 7.

**Figure 8:**
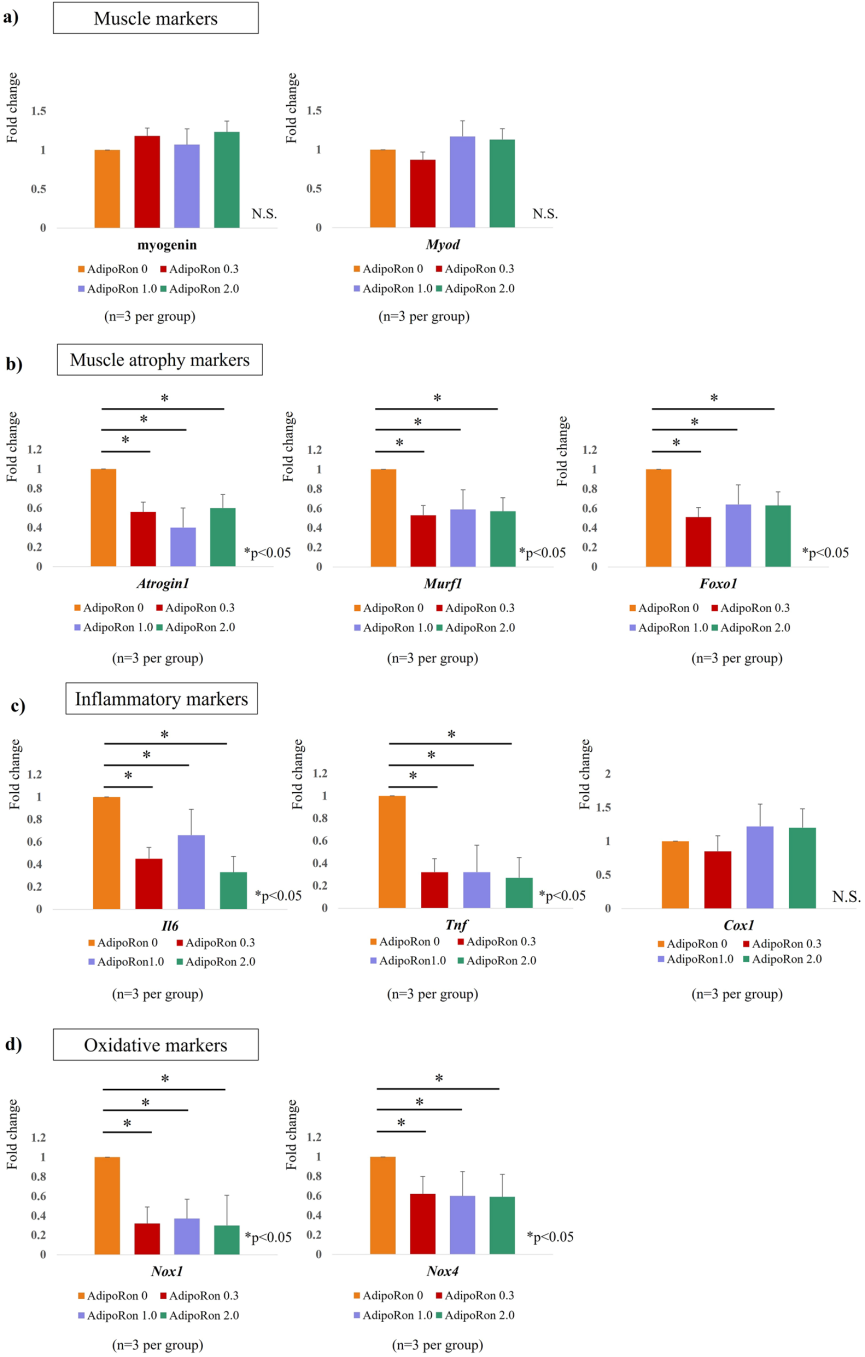
(**a**) comparison of fold change (baseline: AdipoRon 0). AdipoRon treatment did not cause a significant difference in the expression of muscle markers (myogenin, *Myod*), but (**b**) significantly reduced muscle atrophy markers (*Atrogin1*, *Murf1*, *Foxo1*). (**c**) AdipoRon administration significantly reduced the levels of inflammatory cytokines, *Il6* and *Tnf*. The AdipoRon treatment did not affect the level of the pro-inflammatory cytokine, *Cox1*. (**d**) The expression of oxidative markers was also significantly decreased by AdipoRon treatment. No significant differences were seen in groups treated with different AdipoRon doses.

To evaluate the effect of AdipoRon administration in vitro, the nerve-crushed group received AdipoRon at concentrations of 0 μg/ml, 0.3 μg/ml, 1.0 μg/ml, and 2.0 μg/ml, respectively. Although the expression levels of myogenin and *Myod*, the markers of muscle atrophy, were not significantly different after the AdipoRon treatment, the expression levels of *Atrogin1*, *Murf1*, and *Foxo1*, the markers of muscle atrophy, were significantly decreased following AdipoRon treatment, the differences in group which were administered different concentrations were not significant. In terms of inflammatory cytokines, *Il6* and *Tnf* were significantly downregulated after AdipoRon treatment. AdipoRon treatment did not bring about any significant changes in *Cox1* expression. The expressions of *Nox1* and *Nox4*, which are involved in oxidative stress, were significantly decreased by AdipoRon treatment. The groups that received different doses did not show significant differences (Fig. 8).

## Discussion

In this study, we used a nerve-crushed rat model to verify the hypothesis that an imbalance in adipokine secretion owing to fatty degeneration associated with muscle atrophy triggers an inflammatory response. In the nerve-crushed group, the cross-sectional area of the muscle was decreased, fatty infiltration was observed by oil red staining, and the expression of adipogenic differentiation markers was increased. This was accompanied by decreased expression of *Adipoq* in the skeletal muscle and increased levels of inflammatory cytokines and *Nox*, which are involved in oxidative stress. However, the expression of adiponectin receptor, AdipoR1, was not significantly different between the control and nerve-crushed groups. It was also observed that in vitro agonist treatment significantly improved inflammatory response and increased cellular viability. To the best of our knowledge, there is no report on the effect of AdipoRon administration on muscle atrophy. In this in vitro study, AdipoRon administration showed anti-oxidant, anti-inflammatory, and anti-apoptotic effects.

Reportedly, muscle atrophy accompanied by the fatty degeneration of muscles is clinically observed after nerve injury or tendon rupture^4^. However, despite recent improvements in nerve and tendon repair techniques, chronic inflammation and muscle atrophy associated with fatty degeneration still limit postoperative function^6,14^. Muscle atrophy occurs when the breakdown of proteins in muscle cells exceeds their synthesis, resulting in a decrease in muscle mass^15^, and reportedly, inflammatory cytokines and oxidative stress enhance this muscle degradation process.

In the skeletal muscle, myokines secreted by the muscle and adipokines secreted by the white adipose tissue balance the metabolism^16^. Among these cytokines, IL6 and TNF are considered inflammatory cytokines, while adiponectin is considered an anti-inflammatory cytokine^17–19^. Although adiponectin was originally identified as a secretory protein in adipose tissue, it is now known to be expressed in the skeletal muscle^10^. Adiponectin in skeletal muscle has potent anti-inflammatory, antioxidant, and anti-apoptotic effects, as well as a significant inverse correlation with pro-inflammatory cytokines, like IL6 and TNF^20^. These adipokines are tightly regulated to maintain homeostasis in the skeletal muscle^16,21^; however, when adipose tissue proliferates under non-physiological conditions, such as is the case with obesity and degeneration, the adipokine secretion balance is disturbed^22^. Thus, the adipocytes that infiltrate skeletal muscle owing to the degeneration caused by trauma or tendon rupture (ectopic fat) possibly disrupt adipokine homeostasis, resulting in chronic pain^23^. Under such conditions, atrophied muscles with ectopic fat tissue secrete low amounts of adiponectin^23^, and the catabolism induced by TNF is particularly enhanced^24,25^. In addition, NOX-mediated activation of reactive oxygen species causes oxidative stress, which induces apoptosis via the FoxO1/MuRF-1/Atrogin-1 signaling pathway and contributes to muscle atrophy^26,27^. Adiponectin is thought to play an important role in antagonizing these inflammatory and oxidative stresses^20,28^.

Two subtypes of adiponectin receptors (AdipoR1 and AdipoR2) have been identified via complementary DNA expression cloning^29^. Specifically, AdipoR1 is predominantly expressed in skeletal muscles, while AdipoR2, which functions as a major receptor for adiponectin in vivo, is predominantly expressed in the liver^28^. Additionally, AdipoR1 activates the activated protein kinase (AMPK) pathway, which promotes fatty acid burning and reduces inflammation^30,31^. Even though the anti-inflammatory and antioxidant effects of adiponectin via AdipoR1 have been evaluated in some previous studies^32^, reports on the expression of adiponectin and AdipoR1 in atrophied muscles are limited. In this study, the fluorescence immunostaining of muscle samples from the control group showed that adiponectin and its receptor, AdupoR1, were expressed along the fascia. Conversely, the nerve-crushed group showed a significant decrease in adiponectin expression, even though the AdipoR1 expression was maintained. There was no significant difference in the expression of AdipoR1 between the control group and the nerve injury group. These results suggest that AdipoRon administration is useful for correcting the imbalance of adipokines after muscle atrophy. Furthermore, previous reports have demonstrated that it shows anti-inflammatory and anti-fibrotic actions in the liver as well as anti-inflammatory actions in the myocardium^33,34^. In this study, real-time PCR showed decreased *Tnf* and *Il6* expression owing to AdipoRon treatment. However, this treatment had no effect on *Cox1* expression. Therefore, the balance between TNF and IL6 as inflammatory cytokines and the secretion of adiponectin is important for the maintenance of skeletal muscle homeostasis. The expression of muscle atrophy markers and oxidative markers also showed a significant decrease following AdipoRon treatment. Furthermore, the anti-inflammatory and anti-apoptotic effects of adiponectin are expected to be sufficient to maintain the cytokine balance, even at low concentrations given at high concentrations (20 μM). AdipoRon administration to myocytes rather inhibits differentiation into myotubular cells, resulting in a decrease in muscle mass^35^. AdipoRon treatment decreased inflammatory, oxidative, and degenerative markers and increased cellular activity in the nerve-crushed group. These results suggest that AdipoRon has anti-inflammatory, antioxidant, and anti-apoptotic effects in vitro. The doses of AdipoRon used in this study were based on previous reports^36,37^. No cytotoxicity was observed within the range of doses administered. In addition, there was no significant difference in the effect of AdipoRon dose concentration. When examined in light of previous reports^35,36^, this may be due to saturation of AMPK pathway activation. Furthermore, atrophied muscles were used in this study, which may have lowered the response threshold to AdipoRon stimulation. The saturation of the AMPK pathway after AdipoRon administration was thought to lead to a concentration-independent response of anti-inflammatory and antioxidant effects. Therefore, administration of AdipoRon at a low dose concentration may be an effective treatment option for fatty degeneration-associated chronic inflammation and muscle atrophy.

This study had several limitations. The first is that the evaluation period was limited to a single time point (the subacute phase). This implies that the evaluation of the chronic phase needs to be considered in the future. Second, in this study, we evaluated adiponectin receptor agonists only in vitro. However, in vivo expression of adiponectin receptors was observed in the nerve-crushed group, suggesting that the administration of adiponectin may be effective. Therefore, in the future, it would be necessary to examine the dosage concentrations as well as other factors. Further research on the role of AdipoRon in chronic inflammation and muscle atrophy is also required. In vivo evaluation of the effect of AdipoRon on the activation of the AMPK pathway in injured muscle may lead to further confirmation of the therapeutic effect. Third, the present study did not measure differences in the isoforms of adiponectin in gastrocnemius muscle. Since adiponectin physiologically forms multimers^10^, further studies investigating isoforms are needed. Finally, adipokines from atrophied muscle and infiltrated fat cannot be strictly evaluated separately. The balance of adipkines in muscle is important, and correcting the imbalance is expected to prevent chronic pain and degeneration.

In this study, we observed muscle atrophy and fatty infiltration accompanied by a decreased expression of adiponectin which had anti-inflammatory and anti-apoptotic effects in the nerve-crushed group. Conversely, the secretion of inflammatory cytokines increased. This cytokine imbalance possibly resulted in chronic pain owing to the induction of apoptosis and inflammation in the muscle cells. Even though adiponectin expression was decreased in the nerve-crushed group, the expression of AdipoR1 was maintained. Furthermore, the in vitro administration of the adiponectin receptor agonist showed anti-inflammatory and anti-apoptotic effects, suggesting that the control of adipokines via the local administration of AdipoRon could lead to the prevention of fatty degeneration-associated chronic pain.

## Materials and methods

### Animal model

This study was approved by the animal research committee of the Department of Orthopedic Surgery, Graduate School of Medicine, Kobe University, Kobe, Japan. All experiments on animals were conducted under the approval and guidance of the Animal Care and Use Committee of our institution. The experiments were conducted in accordance with the ARRIVE guidelines. Twenty-four SD rats (12-week-old) with a mean weight of 250 g were used in this study (CLEA Japan, Inc., Tokyo, Japan).

### Surgical procedure

All surgical interventions were performed under sterile conditions, with isoflurane, the intraperitoneal injection of pentobarbital sodium (50 mg/kg; Kyoritsu Seiyaku, Tokyo, Japan), and the subcutaneous injection of lidocaine (2.5 mg/kg, Xylocaine®; AstraZeneca, London, UK) at the surgical site as anesthesia.

The skin of the rats was incised at the right hind limbs, and the vastus lateralis and biceps femoris muscles were separated to expose the sciatic nerve. To establish the sciatic nerve injury model, the sciatic nerve was clamped using hemostatic forceps for one minute at a proximal distance of 5 mm from the bifurcation point of the peroneal and tibial nerves (Fig. 1a)^13^. Twelve rats were included in the nerve-crushed group. For the control rats, only a skin incision was made on the hind limbs. The skin incision was closed with a 4–0 nylon suture, and 4 weeks after the surgery, gastrocnemius muscle samples were harvested and analyzed (Fig. 1b).

### Cell culture

After muscle weight measurement, the muscles were minced and treated with 0.4% collagenase for 2 h. After this collagenase treatment, the cells were washed with phosphate-buffered saline (PBS) and centrifuged at 1200 rpm for 5 min. Thereafter, the cells were plated in 100-mm diameter culture dishes and cultured in a monolayer mode using Dulbecco's modified eagle medium (DMEM, HyClone, Logan, UT, USA) mixed with 10% fetal bovine serum (FBS, Cansera, Rexdale, Ontario, Canada), 100 U/ml penicillin, and 100 μg/ml streptomycin. The cells were then evaluated after 2–3 passages and group comparisons were performed.

### Protocol for in vitro AdipoRon administration

Myocytes corresponding to the nerve-crushed group were treated with AdipoRon (AdipoGen Life Sciences, Liestal, Switzerland)^36^, a small molecule adiponectin receptor (AdipoR) agonist. Specifically, the AdipoRon was first dissolved in DMSO to obtain a 2 mM stock solution. Thereafter, it was administered at concentrations of 0.3, 1.0, and 2.0 μg/ml, as previously reported^36,37^, and the differences in the efficacy of this treatment among the treatment groups were determined. Specifically, cell viabilities at 24 h after treatment administration were compared using the WST assay, and the expression levels of *Tnf*, *Il6*, and *Cox1* (inflammatory markers) and myogenin and *Myod* (muscle markers) and *Murf1* and *Atrogin1* (muscle atrophy markers) were evaluated via real-time PCR.

### Evaluation method

#### Cell morphology evaluation

After three passages, 1.0 × 10^5^ cells were seeded into 12-well plates, and the cell morphologies were evaluated via HE staining after 48 h.

#### Cell viability (cell proliferation assay)

Cell viability was measured via a WST assay using a cell counting kit-8 (Dojindo, Kumamoto, Japan). A total of 5,000 cells were seeded into each well of a 96-well-plate and cultured in a DMEM medium for 48 h.

#### Fluorescent immunostaining

The expression level of intracellular adiponectin in myocyte samples from the different groups was detected using an anti-adiponectin polyclonal antibody (NB100-65810F; Bios, Boston, MA, USA). The antibodies, which were used at a dilution ratio of 1:100, were incubated with myocytes (5 × 10^4^) for 60 min in the dark at 37 °C. Thereafter, they were washed twice with PBS and nuclear staining was performed using DAPI solution for 10 min. The percentage of stained cells was then observed using a fluorescence microscope (BZ-8000 confocal microscope; Keyence, Osaka, Japan). For quantification, the number of DAPI-positive and Adiponectin-stained cells in five fields of view (0.75 mm × 1.0 mm) on each slide was counted, and the ratio of the mean values was calculated. The measurements were performed on randomly selected areas by two investigators who were blinded to each other.

#### Histological examination

The weight ratio of the gastrocnemius muscle at the affected side to that at the healthy side was measured. Specifically, gastrocnemius muscle samples were harvested and embedded in an optimal cutting temperature (OCT) compound (Sakura Finetek USA, Inc., Torrance, CA), and stored at −80 °C for histochemical and immunohistochemical staining. Specifically, the gastrocnemius muscle samples in the OCT blocks were sectioned serially to have a thickness of 6 μm. Thereafter, they were was mounted on a silane-coated glass slide, and air-dried for 1 h before fixation with 4% paraformaldehyde at 4 °C for 5 min. The tissue sections were then stained with HE to observe the histological differences between the muscle samples from the control and nerve-crushed groups. Fat droplets were also stained with Oil Red-O solution (Mutoh Pure Chemical, Tokyo, Japan) to evaluate fat infiltration into the muscle. Gastrocnemius muscle was frozen using isopentane cooled with liquid nitrogen and stored at −80 °C until needed. As described in the previously published protocol^38^, the muscles were cryosectioned at a thickness of 10 μm and fixed in 4% paraformaldehyde. The sections were then stained with Oil Red-O and counterstained with hematoxylin.

For immunofluorescence staining, adiponectin antibody and AdipoR1 antibody were used at a dilution ratio of 1:100; the staining was performed at 25 ℃ room temperature for 1 h. To ensure nuclear staining, the DAPI solution was applied for 5 min. All sections were visualized using a fluorescence microscope (BZ-8000 confocal microscope; Keyence). Furthermore, the number of positively stained cells was counted in five randomly selected fields (250 × 250 mm).

#### Real-time PCR

Myocytes from both groups were seeded in 12-well culture plates at a density of 1.0 × 10^5^ cells/well and cultured in DMEM for 48 h. Thereafter, total RNA was extracted using an RNeasy Mini Kit (QIAGEN, Valencia, CA, USA) according to the manufacturer’s protocol. Furthermore, oligo (deoxythymidine)-primed first-strand cDNA was synthesized using a High Capacity cDNA Transcription Kit (Applied Biosystems, Foster City, CA, USA), and quantitative real-time PCR was performed in a 20 μl reaction mixture using the SYBR Green Master Mix reagent (Applied Biosystems) on an ABI Prism 7500 sequence detection system (Applied Biosystems). The PCR conditions were as follows: 1 cycle at 95 °C for 10 min, followed by 40 cycles at 95 °C for 15 s, and 40 cycles at 60 °C for 1 min. The messenger RNA (mRNA) expression levels of *Adipoq* were evaluated. As muscle-related markers, the expression levels of anabolic markers, myogenin and *Myod*, and muscle atrophy markers, *Atrogin1*, *Murf1*, and *Foxo1* were also monitored. Additionally, the mRNA expression levels of *Il6*, *Tnf*, and *Cox1* were analyzed as pro-inflammatory cytokines, *Nox1* and *Nox4* were analyzed as oxidative markers, and *Pparg* and *Cebpa* were used as markers of adipose degeneration.

The primer sequences are shown in Table 1. The relative expression levels of the genes were determined using the DD-Ct method and normalized to *Gapdh*.

**Table 1:** Primers for real-time PCR.

Gene	Forward primer (5' to 3')	Reverse primer (5' to 3')
Adipoq	AATCCTGCCCAGTCATGAAG	CATCTCCTGGGTCACCCTTA
Il6	TCCTACCCCAACTTCCAATGCTC	ACCCAGAGCGTATCATCCTTCAC
Tnf	AAATGGGCTCCCTCTCATCAGTTC	CCAACTGACTTTGAGCCAACGAG
Cox1	TGCCAGTATTAGCAGCAGGT	GAATTGGGTCTCCACCTCCA
Nox1	CTTCCTCACTGGCTGGGATA	TGACAGCATTTGCGCAGGCT
Nox4	AGTCAAACAGATGGGATA	TGTCCCATATGAGTTGTT
Pparg	TGTGGACCTCTCTGTGATGG	CATTGGGTCAGCTCTTGTGA
Cebpa	CCCGATGAGCAGCCACCTCCA	TACCCCGCAGCGTGTCCAGT
Myogenin	CCTTGCTCAGCTCCCTCA	TGGGAGTTGCATTCACTGG
Myod	GGAGACATCCTCAAGCGATGC	AGCACCTGGTAAATCGGATTG
Atrogin1	GAACAGCAAAACCAAAACTCAGTA	GCTCCTTAGTACTCCCTTTGTGAA
Murf1	TGTCTGGAGGTCGTTTCCG	ATGCCGGTCCATGATCACTT
Foxo1	GAGGTGCAATGTGGGAGAAT	TTGAATGAAATGGCAAAGCA
Gapdh	TGGCCTCCAAGGAGTAAGAAAC	GGCCTCTCTCTTGCTCTCAGTATC

### Statistical analysis

All data are expressed as mean values ± standard deviations. Cell viability and real-time PCR results were expressed as n-fold differences relative to the baseline control at the corresponding time point. Student’s t-test was performed to compare two groups, and two-way ANOVA and Tukey's posthoc test were used to compare two or more AdipoRon treatment groups. Results with *p* < 0.05 were considered statistically significant. The data were analyzed using SPSS v23.0 (IBM Corporation, Armonk, NY, USA).

## Data availability statement

The data presented in this study are available on request from the corresponding author. The data are not publicly available because of confidentiality issues.

## Supplementary Information

Supplementary Information.
